# Delayed Impairment of Hippocampal Synaptic Plasticity after Pentylenetetrazole-Induced Seizures in Young Rats

**DOI:** 10.3390/ijms232113461

**Published:** 2022-11-03

**Authors:** Tatyana Y. Postnikova, Alina M. Trofimova, Maria V. Zakharova, Olga I. Nosova, Alexey R. Brazhe, Dmitry E. Korzhevskii, Alexey V. Semyanov, Aleksey V. Zaitsev

**Affiliations:** 1 Sechenov Institute of Evolutionary Physiology and Biochemistry of RAS, Saint Petersburg 194223, Russia; 2 Institute of Experimental Medicine, Saint Petersburg 197022, Russia; 3 Faculty of Biology, Moscow State University, Moscow 119234, Russia; 4 Shemyakin-Ovchinnikov Institute of Bioorganic Chemistry, Russian Academy of Sciences, Moscow 117997, Russia; 5 Department of Clinical Pharmacology, Sechenov First Moscow State Medical University, Moscow 119435, Russia

**Keywords:** long-term potentiation, NMDA receptor, mGluR1, astrocyte, hippocampus

## Abstract

Data on the long-term consequences of a single episode of generalized seizures in infants are inconsistent. In this study, we examined the effects of pentylenetetrazole-induced generalized seizures in three-week-old rats. One month after the seizures, we detected a moderate neuronal loss in several hippocampal regions: CA1, CA3, and hilus, but not in the dentate gyrus. In addition, long-term synaptic potentiation (LTP) was impaired. We also found that the mechanism of plasticity induction was altered: additional activation of metabotropic glutamate receptors (mGluR1) is required for LTP induction in experimental rats. This disturbance of the plasticity induction mechanism is likely due to the greater involvement of perisynaptic NMDA receptors compared to receptors located in the core part of the postsynaptic density. This hypothesis is supported by experiments with selective blockades of core-located NMDA receptors by the use-dependent blocker MK-801. MK-801 had no effect on LTP induction in experimental rats and suppressed LTP in control animals. The weakening of the function of core-located NMDA receptors may be due to the disturbed clearance of glutamate from the synaptic cleft since the distribution of the astrocytic glutamate transporter EAAT2 in experimental animals was found to be altered.

## 1. Introduction

Prolonged recurrent seizures cause serious disturbances in the hippocampus, including neurodegeneration and neurogenesis, axonal sprouting and dendrite remodeling, inflammatory cell invasion, gliosis, angiogenesis, and changes in the extracellular matrix [1,2]. Since the hippocampus plays a key role in memory consolidation [3], such pathological changes lead to the impairment of cognitive functions, especially learning and memory [4,5,6]. The effects of seizures on morphological changes in the developing hippocampus and the functional consequences are poorly understood and largely contradictory [7,8,9].

Pentylenetetrazole (PTZ), a GABA_A_ receptor antagonist [10,11], causes myoclonic seizures and is often used to study the consequences of single episode of seizures [12,13] and to test antiepileptic drugs [14]. The effect of PTZ depends on the age of the animals; generalized tonic-clonic seizures are more typical for rats under three weeks of age, and clonic seizures of facial and forelimb muscles are more characteristic for older rats [15].

The seizure outcomes in young and adult animals are also different. In adult animals, a single episode of PTZ-induced seizures usually does not result in significant neuronal loss in the brain [2,16]; however, it can cause the rapid emergence of dark neurons [17,18], an increase in the number of proliferating cells in the dentate gyrus and the subventricular zone [16], ultrastructural changes in some neurons, glial cells, and synapses in the CA1 field of the hippocampus [19]. Subsequently, one episode of PTZ-induced seizures in adult (two-month-old) male Wistar rats is accompanied by slowly developing cognitive decline [20].

PTZ-induced seizures in 10-day-old (P10) rats resulted in significant neuronal death in the dentate gyrus, whereas neuronal loss did not reach a statistically significant level at P21 [18]. In animals receiving PTZ at P18, another research group revealed cell loss only in the CA3 area of the hippocampus and in the hilus of the dentate gyrus, while in the CA1 area of the hippocampus, the dentate gyrus remained intact [21].

Even a single episode of seizures at P21 can have both short-term and long-term effects on the functioning of neural networks in the hippocampus. In our previous study, we found that the next day following PTZ-induced seizures, pyramidal cells in the hippocampus had increased input resistance and started firing at a lower level of depolarization as compared with the control. Basal excitatory synaptic transmission was reduced in CA3-CA1 synapses, thus preventing the propagation of excitation through neural networks [22]. Within the first week after PTZ-induced seizures, we observed impaired long-term synaptic plasticity in the rat hippocampus [23]. Moreover, NMDA-dependent LTP was suppressed, and LTP transiently switched to the mGluR1-dependent form [23].

The purpose of this work was to study the delayed impairment of hippocampal synaptic plasticity after pentylenetetrazole-induced seizures in young rats

## 2. Results

### 2.1. PTZ-Induced Seizures Resulted in a Decrease in the Number of Neurons in CA1 and CA3 Areas and Hilus of the Hippocampus

**Figure 1:**
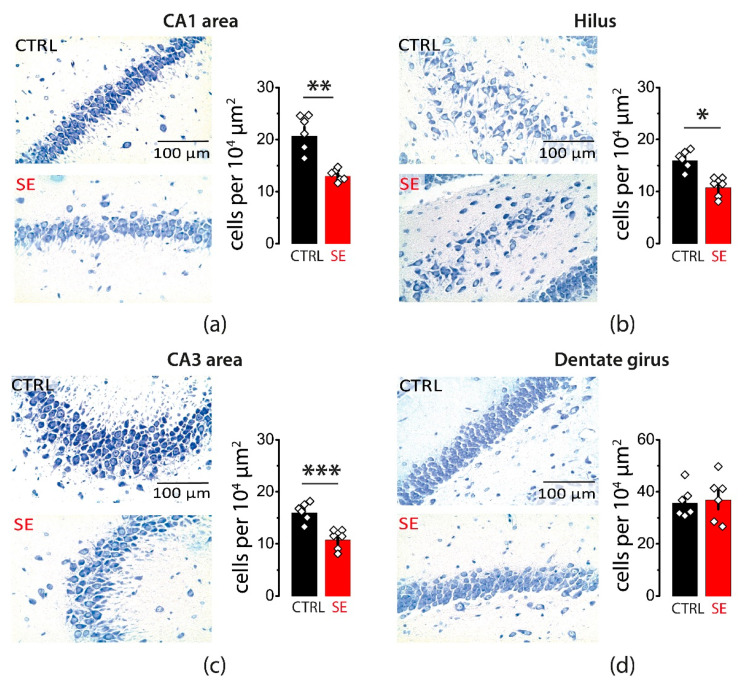
Neurodegeneration in the hippocampal areas 30 days after PTZ-induced seizures. Nissl staining showing neurodegeneration in the *str. pyramidale* of CA1 (**a**), CA3 (**b**), and hilus (**c**). No significant neurodegeneration was observed in the granular cell layer of the dentate gyrus (**d**). Right: illustrations of stained cells in control (top) and post-SE rats (bottom). Left: the summary data from several animals. The cells were counted along the cell layers and normalized to the length of the layers. The circles show values in individual rats. The bars with error bars are means ± standard errors of the means (SEMs). Each rhombus represents a value obtained in an individual animal. *t*-test * *p* < 0.05, ** *p* < 0.01 *** *p* < 0.001.

Whether a single episode of early-onset seizures causes neurodegeneration remains controversial. Therefore, we evaluated potential neuronal loss in various areas of the hippocampus one month after PTZ-induced seizures. Nissl staining confirmed cell loss in pyramidal layers of three hippocampal regions: CA1, CA3, and the hilus (CA1: 20.6 ± 1.7 cells per 10^4^ μm^2^ in control, *n* = 6; 12.9 ± 0.5 cells per 10^4^ μm^2^ after SE, *n* = 6; *t*-test = 4.4, *p* < 0.01; CA3: 26 ± 3 cells per 10^4^ μm^2^ in control, *n* = 6; 16.8 ± 1.3 cells per 10^4^ μm^2^ after SE, *n* = 6; *t*-test = 2.7, *p* = 0.02; Hilus: 15.9 ± 0.7 cells per 10^4^ μm^2^ in control, *n* = 6; 10.7 ± 0.7 cells per 10^4^ μm^2^ after SE, *n* = 6, *t*-test = 5.0, *p* < 0.001; Figure 1a–c). No significant changes in the cell density were observed in the granular cell layer of the dentate gyrus (35.6 ± 2.4 cells per 10^4^ μm^2^ in control, *n* = 6; 36.8 ± 3.5 cells per 10^4^ μm^2^ after SE, *n* = 6; *t*-test = 0.3, *p* = 0.77; Figure 1d).

### 2.2. LTP Was Attenuated in the CA1 Hippocampal Area 30 Days after PTZ-Induced SE

**Figure 2:**
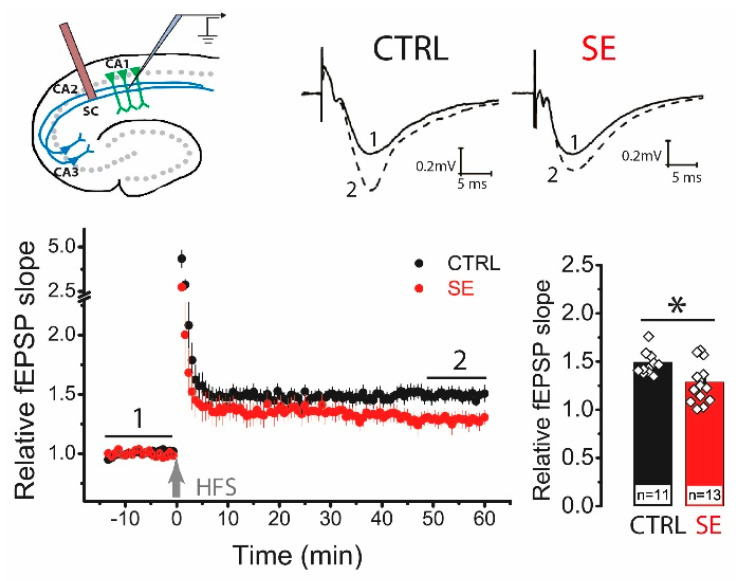
LTP is attenuated in the CA1 hippocampal area 30 days after PTZ-induced seizures. The schema above shows the positions of electrodes in the hippocampus. At the right are representative examples of field postsynaptic potential (fPSP) before (1) and 60 min after (2) HFS in control (CTRL) and post-SE (SE) groups. The diagram below shows the normalized slope of fPSP in control and post-SE groups before and after HFS. The bar graph illustrates the differences in LTP value between groups. All data are presented as a mean ± SEM. Each rhombus represents a value obtained in an individual animal. Student’s *t*-test: * *p* < 0.05.

Next, we analyzed the delayed effect of PTZ-induced seizures on the LTP value in the CA3-CA1 synapses of the rat hippocampus. High-frequency stimulation (HFS) protocol induced significant LTP in the hippocampal slices from control animals (1.49 ± 0.05, *n* = 11), while LTP was significantly reduced in post-SE rats (1.29 ± 0.07; *n* = 13; unpaired *t*-test, *t* = 2.36, *p* < 0.05; Figure 2).

To determine whether the locus of synaptic plasticity induction changed, we compared the paired ratio of field postsynaptic potential (fPSP) amplitudes before and after the application of the HFS protocol. In control animals, the ratio of amplitudes did not change after HFS (baseline: 1.42 ± 0.04; LTP: 1.38 ± 0.06, *n* = 11; paired *t*-test = 1.06, *p* = 0.32). In the post-SE group, no changes in the amplitude ratio were observed either (BL: 1.32 ± 0.04; LTP: 1.32 ± 0.03; *n* = 14; paired *t*-test, *t* = 0.01, *p* = 0.99). No changes in the paired amplitude ratio indicate a postsynaptic locus of plasticity induction in both groups [24,25].

### 2.3. The Impact of Perisynaptic NMDA Receptors on Synaptic Plasticity Is Enhanced in Post-SE Rats

The weakening of LTP can be caused by the disruption of NMDA receptor signaling [26]. These disturbances, in turn, may be triggered by a decrease in the number of NMDA receptors, a change in their subunit composition, or their localization on the postsynaptic membrane.

Receptors localized perisynaptically are thought to be less effective in producing LTP and may even contribute to LTD production [27]. The contribution of extra- and perisynaptic NMDA receptors can be indirectly assessed by applying the use-dependent blocker MK-801 [28]. Since MK-801 blocks the NMDA receptor pore, initial activation and opening of the channel pore are required [29]. It is assumed that low-intensity stimulation activates receptors located mainly in the core part of postsynaptic density (PSD), opposing the presynaptic release site [30]. Thus, MK-801 blocks NMDA receptors located in the PSD mainly, while extra- and perisynaptic receptors remain unblocked for some time. We used this paradigm and compared the effects of MK-801 (10 μM) and the competitive antagonist AP5 (50 μM), which affects all NMDA receptors, on synaptic plasticity. The antagonists were in the bath throughout the experiment.

**Figure 3:**
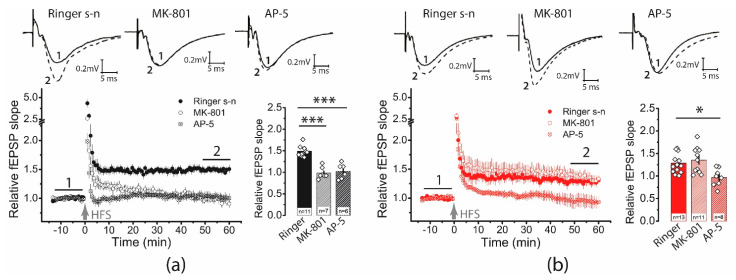
The NMDAR-dependent mechanism of LTP induction is weakened following PTZ-induced SE. On the left, diagrams show the normalized slope of fPSP in control (**a**) and post-SE (**b**) groups. On the right side of each panel are bar graphs (mean ± SEM) showing the effect of NMDAR inhibition with D-AP5 or MK-801 on LTP value in different groups. Each rhombus represents a value obtained in an individual animal. One-way ANOVA followed by Tukey’s post hoc tests: * *p* < 0.05, *** *p* < 0.001.

In control slices, AP5 and MK-801 completely prevented LTP induction (CTRL: 1.49 ± 0.05, *n* = 11; AP5: 1.02 ± 0.07, *n* = 6; MK-801: 0.97 ± 0.05, *n* = 7; one-way ANOVA: F_2,21_ = 27.76, *p* < 0.001; Figure 3a). In contrast, in the post-SE group, only the application of AP5 inhibited long-term plasticity, while the application of MK-801 had no effect on the LTP value (SE: 1.29 ± 0.07, *n* = 13; AP5: 0.97 ± 0.07, *n* = 8; MK-801: 1.35 ± 0.09, *n* = 11; one-way ANOVA: F_2,29_ = 6.05, *p* < 0.01; Figure 3b).

### 2.4. Subunit Composition of NMDA Receptors Is Unchanged in the Post-SE Group

It has been shown that GluN2A-containing NMDARs are predominantly located in the PSD core and play a major role in the induction of LTP [31,32,33], while GluN2B-containing NMDARs are also located in extrasynaptic regions and regulate LTD or reduce the magnitude of LTP [34,35]. Our previous studies have shown that GluN2B-containing NMDA receptor expression may change during the first week after PTZ-induced seizures [36]. Thus, we can assume that the number of peri- and extrasynaptic GluN2B-containing NMDA receptors increase in the hippocampal synapses, and this, in turn, may cause LTP attenuation. Therefore, we decided to evaluate whether the level of expression of GluN2A and GluN2B subunits changes, as well as how the application of the GluN2B selective antagonist ifenprodil (3 μM) affects LTP induction.

**Figure 4:**
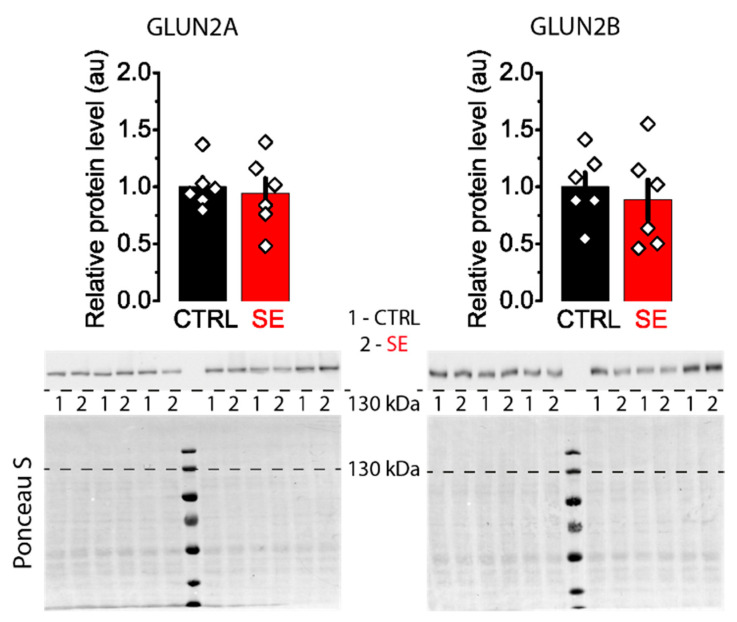
Representative Western blots images of NMDA receptor subunits GluN2A and GluN2B from the dorsal hippocampus of control (CTRL) and experimental (SE) groups. Bar graphs: data presented as mean ± SEM, which were normalized to total protein. *t*-test, *p* > 0.05. Each rhombus represents a value obtained in an individual animal. Images: the upper part shows the chemiluminescent signal, and the lower part shows the Ponceau S.

Western blot assay revealed no significant differences in the expression levels of GluN2A and GluN2B subunits between control and post-SE groups in the dorsal hippocampus (Figure 4).

**Figure 5:**
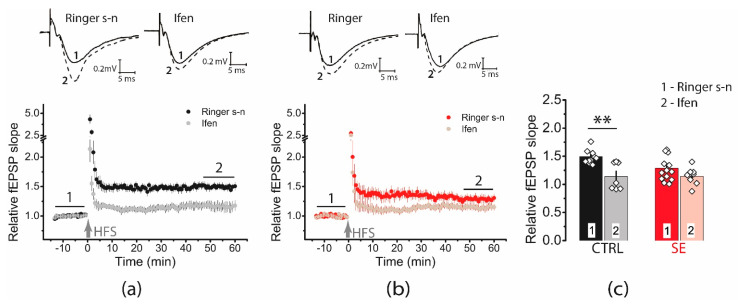
Diagrams showing the effect of ifenprodil (3 μM), a GluN2B selective antagonist, on the normalized slope of fPSP in control (CTRL) (**a**) and post-SE (**b**) groups before and after HFS. (**c**) The bar graphs (mean ± SEM) showing the effect of ifenprodil on LTP values in different groups. Each rhombus represents a value obtained in an individual animal. Two-way ANOVA: F(seizure)_1,35_ = 2.15, *p* = 0.15; F(ifenprodil)_1,35_ = 12.10, *p* < 0.001; F(seizure × ifenprodil)_1,35_ = 2.37, *p* = 0.13 following with Tukey’s post hoc tests: ** *p* < 0.01.

Ifenprodil decreased LTP in the control group (1.49 ± 0.05, *n* = 11 vs. ifenprodil: 1.14 ± 0.09, *n* = 7), but no significant reduction in LTP was observed in the post-SE group (1.29 ± 0.07, *n* = 13 vs. ifenprodil: 1.14 ± 0.05, *n* = 8, Figure 5). Together, these results rather reject the hypothesis about an increase of GluN2B-containing NMDA receptors.

### 2.5. Activation of mGluR1 Is Necessary for Induction of LTP in the Post-SE Group

It is known that the group I mGluRs are preferentially enriched in the perisynaptic domain and seem to be largely excluded from the PSD [30]. Moreover, mGluRs can modulate the activity of nearby NMDA receptors [37]. Since our experimental data suggest a more prominent role of perisynaptic NMDA receptors in the induction of LTP in the post-SE group, we hypothesize that inhibition of mGluRs may affect synaptic plasticity. Previously, we have shown that one day after PTZ-induced seizures, LTP is NMDA-independent and is related to mGluR1 [23]. Therefore, in the present study, we evaluated the role of mGluR1 in LTP induction as well.

**Figure 6:**
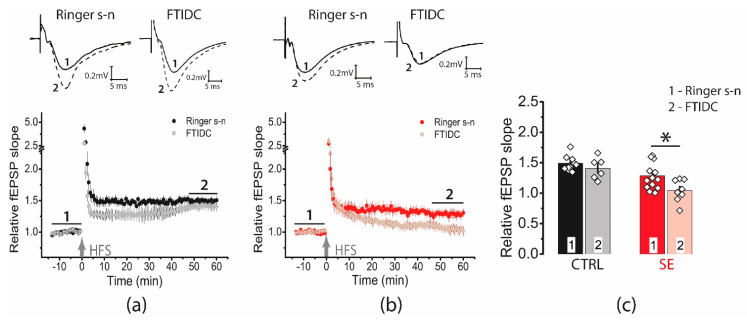
Activation of mGluR1 is necessary for the induction of LTP in the post-SE group. Diagram showing the effect of FTIDS (5 μM) on the normalized slope of fPSP in control (**a**) and post-SE (**b**) groups. (**c**) Bar graphs (means ± SEM) showing the effect of mGluR1 inhibition by FTIDC on LTP magnitude in different groups. Each rhombus represents a value obtained in an individual animal. Two-way ANOVA: F(seizure)_1,34_ = 17.4, *p* < 0.001; F(FTIDC)_1,34_ = 5.7, *p* < 0.05; F(seizure × FTIDC)_1,34_ = 1.3, *p* = 0.26; following with Tukey’s post hoc tests: * *p* < 0.05.

We found that the application of FTIDC (5 μM), a mGluR1 antagonist prevented LTP induction in the post-SE group (1.04 ± 0.06, *n* = 8, Figure 6), whereas the LTP value did not change in the control group (1.40 ± 0.07, *n* = 6). Thus, our data shows that in the post-SE group, LTP induction depends on the cooperative functioning of NMDA receptors and mGluR1, confirming the hypothesis about the important role of perisynaptic glutamate receptors for LTP induction.

### 2.6. Blockade of Glutamate Transporters Has No Significant Effect on Synaptic Plasticity

**Figure 7:**
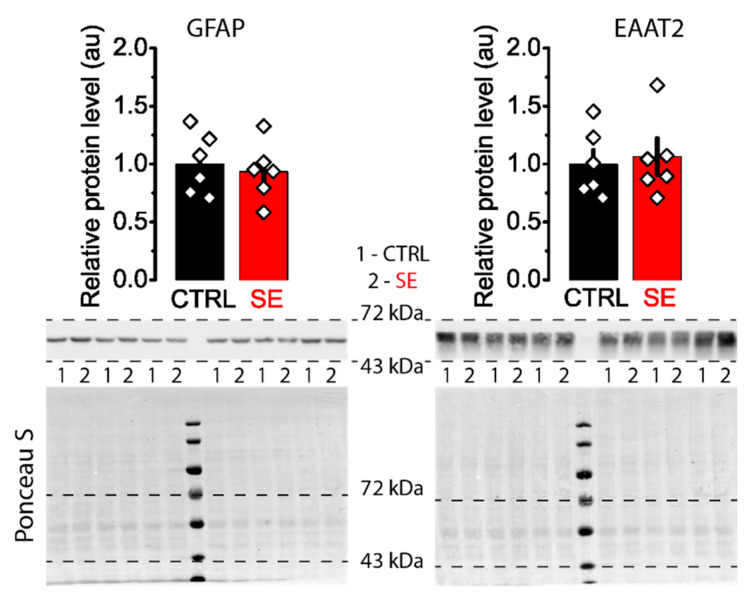
Representative western blots of GFAP and EAAT2 from the dorsal hippocampus of control (CTRL) and experimental (SE) groups. Bar graphs (means ± SEM) illustrate the expression levels of GFAP and EAAT2, which were normalized to total protein. Each rhombus represents a value obtained in individual animals. Images: the upper part shows the chemiluminescent signal, and the lower part shows the Ponceau S.

The change in the role of NMDA receptors located directly in the PSD and perisynaptically in the induction of plasticity may be due to alterations in the clearance of glutamate from the synaptic cleft. It is known that after epileptic seizures and in epilepsy, glutamate transporters can be disrupted [38], and neuron-glial interactions are impaired [39]. Epilepsy is associated with increased GFAP expression. Up-regulation of GFAP induction has been reported in the hippocampal kindling model [40]. The protein level increases in the pilocarpine model of epilepsy in rats [41]. In human subjects diagnosed with epileptic seizures, serum GFAP level is significantly increased compared to healthy controls [42]. Hence, we evaluated the protein expression levels of GFAP and EAAT2 in hippocampal tissue. Western blot analysis revealed no significant changes in the expression levels of these proteins in the post-SE group (Figure 7).

**Figure 8:**
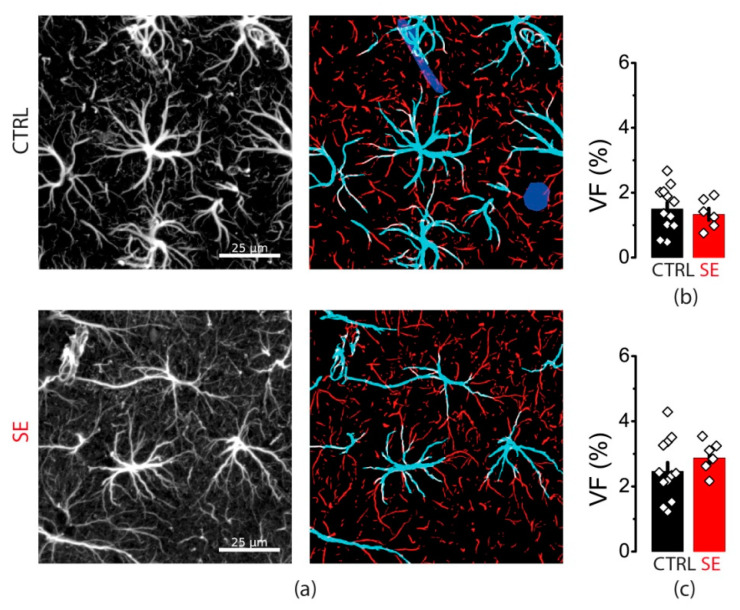
GFAP immunostaining. (**a**) Representative examples of GFAP immunostaining from the dorsal hippocampus of control (CTRL) and experimental (SE) groups. Left: maximal projection of GFAP-stained stack. Right: segmentation to thick (cyan) and thin (red) GFAP-positive processes. Volume fractions (VF) of thick (**b**) and thin (**c**) astrocyte processes. Data presented as mean ± SEM. Each rhombus represents a value obtained in an individual animal. *t*-tests, *p* > 0.05.

Protein expression level does not reveal subcellular protein distribution. Hence, we performed the immunochemical staining of GFAP in slices obtained from control animals and the SE model. We divided all stained GFAP processes into two groups: thick primary processes adjoined to cell soma and more distant thin processes (Figure 8).

There was no difference in the volume fraction of either thick or thin processes between control and PTZ slices (Figure 8). Taken together with Western blot data, this result suggests that there are no changes in GFAP expression or distribution as in human epilepsy or other animal models. However, this result does not exclude that they may occur in the kindling PTZ model with different mechanisms.

**Figure 9:**
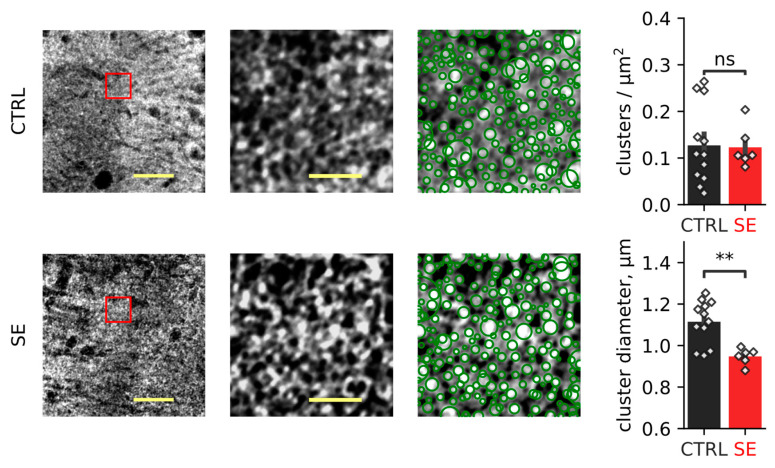
EAAT2 immunostaining. Representative examples of EAAT2 immunostaining from the dorsal hippocampus of control (CTRL) and experimental (SE) groups. Left: maximal projection of EAAT2-stained stack. Scale bar = 25 µm. Middle: Expanded part of the image boxed in the left image. The expansion shows the structural organization of EAAT2, which forms clusters. Scale bar = 5 µm. Right: detection of EAAT2 clusters. Bar graphs show relative EAAT2 cluster density and the mean cluster diameter. Data presented as mean ± SEM. Each rhombus represents a value obtained in an individual animal. *t*-test, ** *p* < 0.01.

In EAAT2 staining, we observed that transporters form bright spots, which we called clusters (Figure 9). These clusters of transporters are typically located in the vicinity of glutamate synapses [43]. We observed a reduction of the mean cluster diameter from 1.11 ± 0.11 to 0.95 ± 0.04 μm (reported as mean ± standard deviation) but not cluster density. These results may indicate that the spatial distribution of EAAT2 is altered in the post-SE group, which may affect glutamate uptake and synaptic plasticity [44]. Therefore, we examined the effect of glutamate transporter blockade on LTP.

Glutamate released during the application of HFS protocols allows prolonged activation of NMDA receptors [45], and it also diffuses out of the synaptic cleft and binds to NMDARs and mGluRs in the peri- or extrasynaptic membrane or at neighboring synapses [46]. EAATs control the degree of glutamate spillover and glutamate access to receptors located at peri- and extrasynaptic sites [44]. Typically, EAATs downregulation leads to a reduction of LTP magnitude and promotes LTD [47,48].

**Figure 10:**
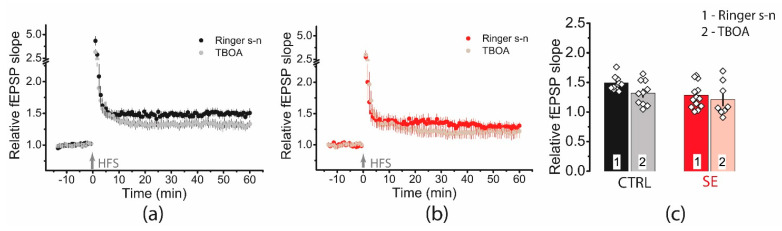
Blockade of glutamate transporters has no significant effect on synaptic plasticity. Diagram showing the effect of TFB-TBOA (70 μM) on the normalized slope of fPSP in control (**a**) and post-SE (**b**) groups. (**c**). Bar graphs (means ± SEM) showing no effect of TFB-TBOA on LTP magnitude. Each rhombus represents a value obtained in an individual animal.

We applied TFB-TBOA (70 μM), which blocks mainly astrocyte transporters EAAT1 and EAAT2 [49]. However, a two-way ANOVA showed no significant effect of transporter blockade on plasticity (F(TBOA)_1,38_ = 3.14, *p* = 0.08) or differences in blocker action between the two groups (F(TBOA × seizure)_1,38_ = 0.47, *p* = 0.49, Figure 10).

## 3. Discussion

Our study showed that a single episode of generalized seizures in three-week-old rats leads to long-term consequences, including neurodegeneration in the hippocampus and impaired synaptic plasticity.

Epileptic seizures significantly affect the functioning of synapses, and alterations have been described at the level of the post- and presynapse, as well as disturbances in the functioning of microglia and astrocytes [50]. One of the most serious consequences of PTZ-induced seizures detected in this study is impaired LTP. In post-SE rats, the magnitude of LTP induced by the HFS protocol was reduced almost twofold compared to the control group. In a previous study, we showed that LTP induction is reduced during a week after PTZ-induced seizures [23], but now we have found that this reduction of LTP lasts for at least a month. The LTP attenuation shown in this work agrees well with other experimental data obtained using various epilepsy models [51,52,53,54,55].

Currently, there is no consensus on the cause of synaptic plasticity impairment following seizures. Since LTP induction is a complex mechanism, disorders of plasticity can be caused by various causes [23,52,56,57,58]. Some studies point to a significant contribution of neurodegenerative changes in brain tissue [59], while other works suggest disturbances in astrocyte function [54] or underline the critical role of neuroinflammation [12].

In this study, we found that not only NMDA receptors but also mGluR1 activation is required for LTP induction in post-SE rats. In contrast, in the control group, mGluR1 inhibition had no effect on LTP value in our experiments. We hypothesized that these changes in the mechanism of LTP induction were due to the prolonged attenuation of NMDA-dependent signaling following seizures. The results of previous studies are generally consistent with this hypothesis. Using c-Fos staining at different time points after PTZ treatment and EEG recordings, it was shown that neuronal activity was maximal at 2 h after injection and diminished at 4 h [60]. One day after PTZ-induced seizures, a decrease in basal excitatory synaptic transmission in CA3-CA1 synapses and a significant increase in the maximum electroshock threshold was detected in rats [22]. A decrease in the amplitude of the field responses in the hippocampal slices was detected even 30 d after PTZ-induced seizures [22].

Therefore, in the post-SE group, enhancement of NMDA-dependent signaling is necessary for LTP induction. Group I mGluRs are known to be involved in the modulation of NMDA receptor activity [61,62]. The effect of mGluRs (activation or inhibition) on NMDA receptors depends on cell type and location. Potentiation of NMDA receptors is mainly observed in dentate gyrus neurons and the CA1 field of the hippocampus, whereas inhibition has been found in cortical neurons and cerebellar cells [30,63]. In the hippocampus, activated group I mGluRs modulate NMDARs simultaneously through Homer- and G-protein-controlled pathways by changing the probability of NMDAR opening [61]. G-protein signaling cascades are the transfer mechanism of this modulation via activation of PLC; at least part of the potentiation is due to a rise in intracellular Ca^2+^ and stimulation of PKC [64]. The second mechanism is a direct molecular link between mGluRs and NMDARs [61,65]. In this case, mGluRs and NMDARs must be closely located on the neuronal membrane: Homer proteins bind to the mGluRs and to the proteins of the Shank family; the Shank proteins, in turn, connect to NMDARs via the protein PSD-95 [61].

The mGluRs must be closely located to NMDARs to efficiently activate them. It was shown that group I mGluRs were preferentially enriched in the perisynaptic domain [30]. Therefore, we believe that perisynaptic NMDARs are involved in LTP induction. Data from our experiments with an irreversible open-channel blocker MK-801 provide indirect evidence in favor of this assertion. We revealed that bath application of MK-801 completely suppresses LTP in the control group, while it has almost no effect on LTP production in brain slices from post-SE rats. It can be assumed that during the test stimulation, only NMDARs located in the core part of PSD are mainly activated and, consequently, blocked. Since during this type of stimulation, glutamate usually does not reach perisynaptic NMDARs, they remain unblocked.

Previous studies have shown that NMDARs with different subunit compositions are unevenly distributed at the core part of PSD and perisynaptic area [66]. While GluN2A-containing NMDARs are localized mainly at the core part, GluN2B-containing NMDARs are more often observed at the perisynaptic part of the membrane [37,38]. Therefore, it was reasonable to hypothesize that selective inhibition of GluN2B-containing NMDARs would have a greater effect on LTP induction in the group of post-SE rats. Therefore, we compared the effect of ifenprodil, a GluN2B selective antagonist, on LTP induction. However, in the presence of ifenprodil, the HFS protocol caused a small increase in responses (approximately 15%) in both groups. Obviously, GluN2B-containing NMDARs play an important role in LTP induction in both groups, but the preferential contribution of GluN2B-containing NMDARs in LTP induction in the group of post-SE rats was not confirmed by this experiment. We additionally compared the expression levels of GluN2A and GluN2B subunits between control and post-SE groups in the dorsal hippocampus but also found no differences.

One of the causes of dysregulation of NMDA-dependent signaling may be impaired clearing of glutamate in the synaptic cleft due to disturbances in neuron-glial relationships. A decrease in EAAT2 levels in the astrocytes of the sclerotic hippocampus has been found in several studies based on clinical and postmortem data in people with temporal lobe epilepsy [67,68], but conflicting data were obtained in animal epilepsy models. The lithium-pilocarpine model showed an increase in EAAT2 mRNA levels in the medial prefrontal cortex and dorsal hippocampus one week after status epilepticus [69]. However, a decrease in EAAT2 expression at the protein level has been shown in another study on the same model [70].

We did not observe a significant change in the protein level of EAAT2, astrocyte-specific glutamate transporter. However, PTZ-induced SE affected the subcellular redistribution of transporters: EAAT2 clusters became smaller, although their spatial density remained unchanged. The dramatic ultrastructural, molecular, and functional remodeling of astrocytes and CA3-CA1 excitatory synapses of the hippocampus has been shown in the kainate rat model of epilepsy [71]. The authors found increased astrocyte ensheathment around both the presynaptic and postsynaptic elements and reduced perisynaptic astrocyte GLT-1 expression [71]. Another possible mechanism is the disruption of glutamate uptake kinetics. Using the repeated low-dose kainate rat model of epilepsy, Takahashi et al. showed a significant decrease in the decay time kinetics of glutamate transport currents recorded in reactive astrocytes, although there was little concomitant change in the total expression of GLT-1 (EAAT2) [72].

In our animal model, redistribution of EAAT2 expression could also affect glutamate uptake and synaptic plasticity [44]. We have not specifically measured glutamate uptake, but we have examined the effects of transporter blockade on plasticity. Our experiments did not reveal changes in LTP that could be explained by impaired glutamate uptake.

Notably, we did not observe a significant increase in GFAP expression at the level of protein or any changes in its subcellular distribution following PTZ-induced SE. This contrasts with previous reports on the up-regulation of GFAP induction in the hippocampal kindling model [40] and protein level increase in the pilocarpine model of epilepsy in rats [41]. Thus, the PTZ model may recruit different cellular and molecular mechanisms in astrocytes. One month after the seizures, we detected a decrease in the number of neurons by about 30% in the hippocampal regions CA1, CA3, and hilus. In various epilepsy models, the hippocampus is one of the structures in which the strongest neuronal loss is observed. However, the most damaged areas of the hippocampus may vary in different models and at different ages of the animals. PTZ-induced seizures in six-week-old rats lead only to the appearance of dark neurons in CA1, CA3b,c, and hilus but do not cause neuronal death [2]. This may be due to the shorter duration and lower severity of PTZ-induced seizures in adolescent rats [15]. In P10 rats, febrile seizures damaged the neurons in the CA1 and hilus areas, while no apparent changes in the number of cells in CA3 and DG were detected [73]. Pilocarpine-induced seizures in three-week-old rats led to a very rapid neuronal death (about 30% of neurons) induced by an excitotoxic effect of glutamate in the hippocampus and dentate gyrus [58,74]. In mice, kainite-induced seizures led to extensive neuronal loss in the CA1 and CA3c regions and in the hilus of the dentate gyrus. In addition, an enlargement of the dentate gyrus with the dispersion of the granule cells was observed [75].

PTZ-induced seizures, unlike pilocarpine-induced seizures or kainate-induced seizures, do not typically lead to the further development of spontaneous recurrent seizures and the development of acquired drug-resistant temporal lobe epilepsy. However, this model, along with other animal models, may be useful for studying the mechanisms of seizure effects on structural and functional alterations in the cortex and hippocampus, including neurodegeneration, axonal sprouting, synaptic reorganization, astrogliosis, changes in synaptic plasticity and neuron-astrocyte interactions, which are the basis of the “neural network hypothesis” of drug-resistant epilepsy [76,77]. Understanding these mechanisms is a necessary basis for the search and development of new pharmacological agents specifically designed for the prevention of epilepsy [78].

## 4. Materials and Methods

### 4.1. PTZ Model of Status Epilepticus

In 3-week-old male Wistar rats, acute seizures were induced by intraperitoneal administration of PTZ (70 mg/kg; Sigma-Aldrich, St. Louis, MO, USA). A characteristic feature of PTZ action on rats at this age is that a single injection causes prolonged convulsions in most animals, which is not common in older rats [15]. The study was conducted only on rats with generalized tonic-clonic seizures lasting at least 45 min. The control rats received a physiological solution.

### 4.2. Brain Slices Preparation

Hippocampal slices were prepared as previously described [23]. Briefly, at the age of 51–55 days, the rats were decapitated, the brain was rapidly removed, and horizontal brain slices (400 μm) were cut using a vibratome HM 650 V (Microm, Walldorf, Germany) in ice-cold artificial cerebrospinal fluid (ACSF), which was aerated with carbogen (95% O_2_ and 5% CO_2_). Composition of ACSF (in mM): 126 NaCl, 2.5 KCl, 1.25 NaH_2_PO_4_, 1 MgSO_4_, 2 CaCl_2_, 24 NaHCO_3_, 10 glucose. The sections were then kept in a water bath at 35 °C for 1 h. After incubation, individual sections were transferred to the experimental chamber.

### 4.3. Field Potential Recordings

Extracellular fPSPs were evoked with a bipolar nichrome electrode placed in the Schaffer collaterals at the CA1–CA2 border and recorded from the CA1 *stratum radiatum* by glass microelectrodes (0.2–1.0 MΩ). Stimulus intensity was chosen so that the amplitude of fPSPs was between 40–50% of the amplitude at which the population spike first appeared. Paired electrical stimuli with an interstimulus interval of 50 ms and a pulse duration of 0.1 ms were delivered every 20 s through the A365 stimulus isolator (World Precision Instruments, Sarasota, FL, USA). Signals were amplified by a Model 1800 amplifier (A-M Systems, Carlsborg, WA, USA), then digitized with ADC/DAC NI USB-6211 (National Instruments, Austin, TX, USA) and stored on a personal computer using WinWCP v5.x.x software (University of Strathclyde, Glasgow, UK). The electrophysiological records were studied using the Clampfit 10.2 program (Axon Instruments, San Jose, CA, USA).

LTP was induced by HFS protocol (3 trains every 20 s, consisting of 100 pulses at 100 Hz). LTP induction was preceded by a 20-min baseline period. Potentiated fPSPs were recorded for 60 min after HFS. LTP was quantified by calculating the ratio of the mean slope of potentiated fPSPs (50–60 min after HFS) to baseline (10 min before HFS).

### 4.4. Western Blot Assay

Rats were decapitated 30 days after PTZ-induced SE. After decapitation, brains were quickly removed, frozen, and stored at −80 °C before dissection. The dorsal area of the hippocampus was dissected at −20 °C using OTF5000 Cryostat Microtome (Bright Instrument, Huntingdon, UK) according to the rat brain atlas [79]. Samples were homogenized in 200 µL of lysis buffer [80] containing 100 mM Tris-HCl pH 8.0, 140 mM NaCl, 20 mM EDTA, 5% SDS, 1X protease inhibitor cocktail (Pierce Protease Inhibitor Tablets; Thermo Fisher Scientific, Wilmington, DE, USA), and 1× phosphatase inhibitor cocktail (Phosphatase Inhibitor Cocktail II; Abcam, Cambridge, UK), and then incubated for 1 h at room temperature and centrifuged at 14,000 ×*g* for 10 min, and the supernatant was used for protein quantification and western blot. Protein concentrations were determined by a modified Lowry assay [81] with BSA as a standard. Protein supernatants were mixed 1:1 with 2× loading buffer (125 mM Tris–HCl pH 6.8, 40% glycerol, 4% SDS, 2.5% β-mercaptoethanol, 0.02% bromophenol blue) and heated at 70 °C for 15 min.

We separated 6 µg of protein using SDS-PAGE [82] in 7% acrylamide gel with Thermo Scientific Page Ruler Prestained Protein Ladder (10–170 kDa; Thermo Fisher Scientific) under 125 V and then transferred to the 45µm nitrocellulose membrane by semi-wet transfer with 1X Power Blotter 1-Step Transfer Buffer (Thermo Fisher Scientific). After transfer, membranes were stained with 0.1% Ponceau (dissolved in 5% acetic acid) and visualized with a ChemiDocMP imager (Bio-Rad, Hercules, CA, USA) for subsequent normalization to total protein. After washing from Ponceau, membranes were blocked in 5% dry milk in PBST for 1 h at room temperature, then incubated in primary antibody solution in PBST containing 0.05% NaN_3_ and rabbit anti-GluN2A (1:1000, ab169873, Abcam), rabbit anti-GluN2B (1:1000, ab65783, Abcam), rabbit anti-EAAT2 (1:3000, ab205248, Abcam) or rabbit anti-GFAP (1:20,000, ab7260, Abcam) overnight at 4 °C. Subsequently, membranes were washed with PBST 5 times followed by incubation with a secondary antibody solution in PBST containing 5% dry milk (1:60,000 Pierce Goat anti-rabbit IgG-HRP, 31460, Thermo Fisher Scientific) at room temperature for 1 h. After 3 washes of 10 min with PBST, proteins were detected with SuperSignal™ West Pico PLUS Chemiluminescent Substrate (Thermo Fisher Scientific) and visualized with a ChemiDocMP imager (Bio-Rad). Images were analyzed using Image Lab 6.0.1 software (Bio-Rad). Protein expression was normalized to the total protein loading of Ponceau–stained membranes [83] following a total protein normalization method (Bio-Rad). The ratio between the optical densities of the specific protein band to the total protein was calculated and normalized to the mean of the control group.

### 4.5. Nissl Staining

The animal brains were removed immediately after decapitation and immersed in a fixing solution of zinc-ethanol-formaldehyde [84]. Then, after dehydration, the brain tissues were embedded in paraffin. Paraffin sections (10 μm) were cut in a coronary plane and stained with Nissl’s method to assess the degree of neurodegeneration. Images of CA1, CA3, hilus, and dentate gyrus were obtained using an × 40 magnification. The neurons were counted in a 100 × 100 μm box positioned in the cell layer using the plugin “Cell counter” for ImageJ (U.S. National Institutes of Health, Bethesda, MD, USA).

### 4.6. Immunocytochemical Method

To detect GFAP and EAAT2 (GLT-1) in astrocytes, we used an immunocytochemical assay. The routinely dewaxed sections were heated in antigen retrieval solution S1700 (Dako, Glostrup, Denmark). Then the sections were incubated in a mixture of primary antibodies of GFAP (1:100; monoclonal mouse antibodies, clone SPM507, E16510; Spring Bioscience, Pleasanton, CA, USA) and glutamate transporters GLT-1 (1:150; polyclonal rabbit antibodies, ab106289; Abcam, Cambridge, UK). The following secondary antibodies were used: monovalent Fab-fragment of anti-rabbit donkey immunoglobulin conjugated with Rhodamine Red™-X (RRX) fluorochrome (1:25, 711-295-152; Jackson ImmunoReaserch, Philadelphia, Pennsylvania, USA) and monovalent Fab-fragment of anti-mouse donkey immunoglobulin conjugated with Cy-2 fluorochrome (1:50, 705-225-147; Jackson ImmunoReaserch, Philadelphia, PA, USA). The sections were mounted in a water-soluble Fluorescence Mounting Medium (Dako, Glostrup, Denmark) and were imaged using an LSM 710 confocal laser microscope (Zeiss, Oberkochen, Germany) with an alpha Plan-Apochromat 100x/1.46 Oil DIC M27 objective, in the z-axis scanning mode in Zen2011 Black. Obtained images were used for further analysis.

### 4.7. Analysis of Immunostaining Data

Immunostaining data was characterized with custom scripts and Jupyter notebooks written in the Python programming language using NumPy, SciPy, and Scikit-Image libraries. GFAP immunostaining quantification relied on volume segmentation to GFAP-positive astrocytic structures and background. The segmentation was done in two steps: intensity-based, followed by Frangi vesselness contrast [85]. Intensity-based segmentation was made by hysteresis thresholding using 3 and 5 standard deviations of image noise as low and high thresholds, correspondingly. The segmentation was then refined with multiscale Frangi vesselness contrast, which enhances anisotropic filamentous structures at a set of spatial scales defined by convolution with a Gaussian kernel with different sigma values. The spatial scales were split into those corresponding to “thin” astrocytic processes and those corresponding to “thick” processes with a boundary of σ = 0.6 μm, chosen to visually separate the two types of processes. Frangi contrast images were obtained as maximum projections across spatial scales within each spatial scale range (0.1–0.6 μm and > 0.6 μm) and binarized to the foreground and background voxels. Volume fractions of thick and thin astrocyte processes, as well as sizes of process skeletons, were collected for each analyzed Z-stack. Because thicker GFAP-positive processes tend to be brighter in the stain, an independent intensity-based separation between larger and small processes was created from a 3-level Otsu thresholding procedure, where the lowest level corresponded to the background, the intermediate level corresponded to dimmer, thin processes, and the highest level separated larger, brighter processes.

Because EAAT2 immunostaining does not reflect any obvious morphology, its quantification differed from that of GFAP and was based on structural image texture analysis algorithms. In essence, we analyzed individual XY planes within each 3D imaged volume using (i) the Laplacian of Gaussian blob detection algorithm to quantify bright EAAT2-positive granules and (ii) the texture separation to granules and ridges (e.g., stringy aggregates of granules) using the shape index algorithm [86]. Specifically, the relative brightness of EAAT2-positive ridges and granules (“caps” in shape index terminology), as well as relative areas taken by ridges and granules and granule radii, were collected as averaged numbers for the XY-planes within each analyzed volume.

### 4.8. Drugs

Antagonists of NMDARs: D-2-amino-5-phosphonovalerate (AP5; 50 μM); (5*R*,10*S*)-(+)-5-methyl-10,11-dihydro-5*H*-dibenzo[*a*,*d*]cyclohepten-5,10-imine (MK-801; 10 µM); selective antagonist of GluN2B-containing NMDARs: 4-[2-(4-Benzylpiperidin-1-yl)-1-hydroxypropyl]phenol (ifenprodil; 3 μM); selective mGluR1 antagonist: 4-[1-(2-fluoropyridin-3-yl)-5-methyltriazol-4-yl]-N-methyl-N-propan-2-yl-3,6 dihydro-2H-pyridine-1-carboxamide (FTIDS; 5 μM); antagonist of glutamate transporters EAAT1 and EAAT2: (2S,3S)-3-[3-[4-(trifluoromethyl) benzoylamino] benzyloxy] aspartate (TFB-TBOA; 70 μM); co-agonists of NMDARs: D-serine (10 μM).

### 4.9. Statistical Analysis

The statistical analysis and graphical representation of the results were performed using OriginPro 8 (OriginLab Corporation, Northampton, MA, USA), Statistica 8.0 (StatSoft Inc., Tulsa, OK, USA), and Sigmaplot 12.5 (Systat Software Inc., Palo Alto, CA, USA). Dixon’s Q test (at a 90% confidence level) was used for the identification and rejection of outliers. The normality of the sample data was evaluated using the Kolmogorov–Smirnov test. The equality of variance was assessed using the Levene median test. Statistical significance was assessed using the nonparametric Mann-Whitney test, parametric Student’s t-test, and ANOVA, as stated in the text. All data are presented as mean ± SEM.

## Data Availability

The data presented in this study are available on request from the corresponding author.
